# Robotic-Assisted Resection of a Giant Sigmoid Colonic Lipoma: A Case Report

**DOI:** 10.7759/cureus.94279

**Published:** 2025-10-10

**Authors:** Yuri Kanaya, Hideyuki Masui, Yusuke Kimura, Motoko Mizumoto, Osamu Takeyama

**Affiliations:** 1 Department of Surgery, Hirakata Kohsai Hospital, Osaka, JPN

**Keywords:** giant colonic lipoma, minimally invasive surgery, robotic surgery, sigmoid colonic lipoma, sigmoidectomy

## Abstract

Colonic lipomas (CLs) are rare, benign non-epithelial tumors composed of adipose tissue. While most CLs are asymptomatic, those exceeding 2 cm can cause symptoms such as abdominal pain, bowel obstruction, intussusception, rectal bleeding, or perforation. Surgical resection is indicated in symptomatic cases or when malignancy cannot be ruled out. Although laparoscopic surgery has been the standard minimally invasive approach, robotic-assisted surgery has emerged as an alternative with enhanced precision and visualization. Herein, we report a rare case of a giant sigmoid CL successfully treated with robotic-assisted colectomy. An 85-year-old man with a history of open distal gastrectomy and chronic heart failure initially presented with rectal mucosal prolapse four years prior. Colonoscopy and MRI identified a submucosal tumor (SMT) measuring approximately 5 cm, diagnosed as a large sigmoid CL. The patient opted for surveillance with MRI every six months, during which no symptoms developed. However, after four years, he experienced a recurrence of rectal mucosal prolapse accompanied by bloody stool, prompting the decision for elective surgery. A robotic-assisted sigmoid resection was performed using a medial approach, preserving the left colic artery while dissecting the superior rectal and sigmoid arteries. The rectum was transected with a 60-mm stapler, and reconstruction was performed using the double-stapling technique. The procedure was completed without complications, and pathological analysis confirmed the diagnosis of a benign submucosal lipoma. The patient was discharged two weeks postoperatively and remained recurrence-free with restored bowel function at an eight-month follow-up. This case illustrates that when CLs grow beyond 2 cm and become symptomatic, surgical resection should be considered, and robotic-assisted colectomy can be a feasible option.

## Introduction

Colonic lipomas (CLs) are relatively rare benign, non-epithelial tumors composed of adipose tissue, with an incidence ranging from 0.2% to 4.4% [1]. Most CLs are asymptomatic and discovered incidentally; however, lesions exceeding 2 cm in size may cause symptoms such as abdominal pain, bowel obstruction, intussusception, bleeding, or perforation [2,3]. They are frequently found in women aged 40 to 70 years with a higher prevalence in the right colon, whereas in men, they are commonly located in the left colon [4-6].

Diagnosis is primarily based on colonoscopy, computed tomography (CT) scans, or magnetic resonance imaging (MRI), and treatment options include surveillance, endoscopic, or surgical resection [1]. Surgery is indicated for symptomatic or large CLs, particularly when malignancy cannot be ruled out. Although laparoscopic surgery has been traditionally the preferred minimally invasive approach, robotic-assisted surgery has emerged as an effective alternative, offering enhanced dexterity, precision, and better visualization in confined spaces such as the pelvic cavity [7]. Here, we present a case of a large sigmoid CL causing recurrent bloody stool, successfully treated by robotic-assisted sigmoid resection.

## Case presentation

The patient was an 85-year-old man with a history of open distal gastrectomy and chronic heart failure. He had presented to a local clinic with a complaint of rectal mucosal prolapse four years prior to his first visit to our department. He received manual reduction for rectal mucosal prolapse and was referred to our hospital’s gastroenterology department for colonic endoscopy screening. Colonic endoscopy revealed an SMT measuring approximately 5 cm in the sigmoid colon, which was further evaluated using MRI and diagnosed as a benign lipoma. The patient opted for careful observation and underwent MRI surveillance every six months, during which no symptoms developed.

**Figure 1:**
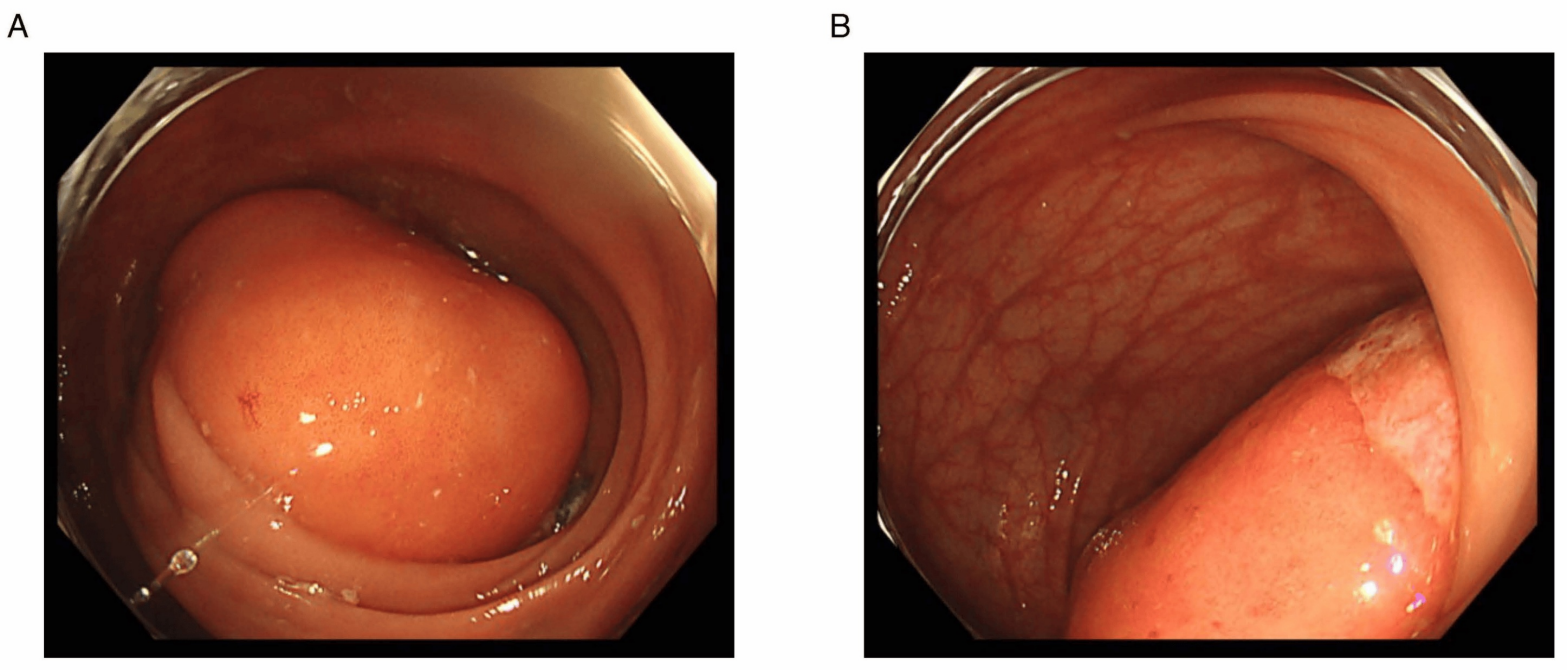
Preoperative colonoscopy findings of the giant sigmoid colonic lipoma (A) A large SMT with a smooth surface is observed in the sigmoid colon. (B) Erosion at the tumor’s apex, suspected as the source of recurrent bloody stool, is evident.

However, four years after his first visit, he returned with a recurrence of rectal mucosal prolapse accompanied by bloody stool, which was again treated by manual reduction. He was admitted for observation, and the bloody stool subsided the next day, allowing for discharge. One week after this event, he revisited our hospital for recurrent bloody stool. A follow-up colonoscopy demonstrated an SMT with erosion at the top of the tumor and occupying the colonic lumen, which was not observed in the prior assessment (Figure 1).

**Figure 2:**
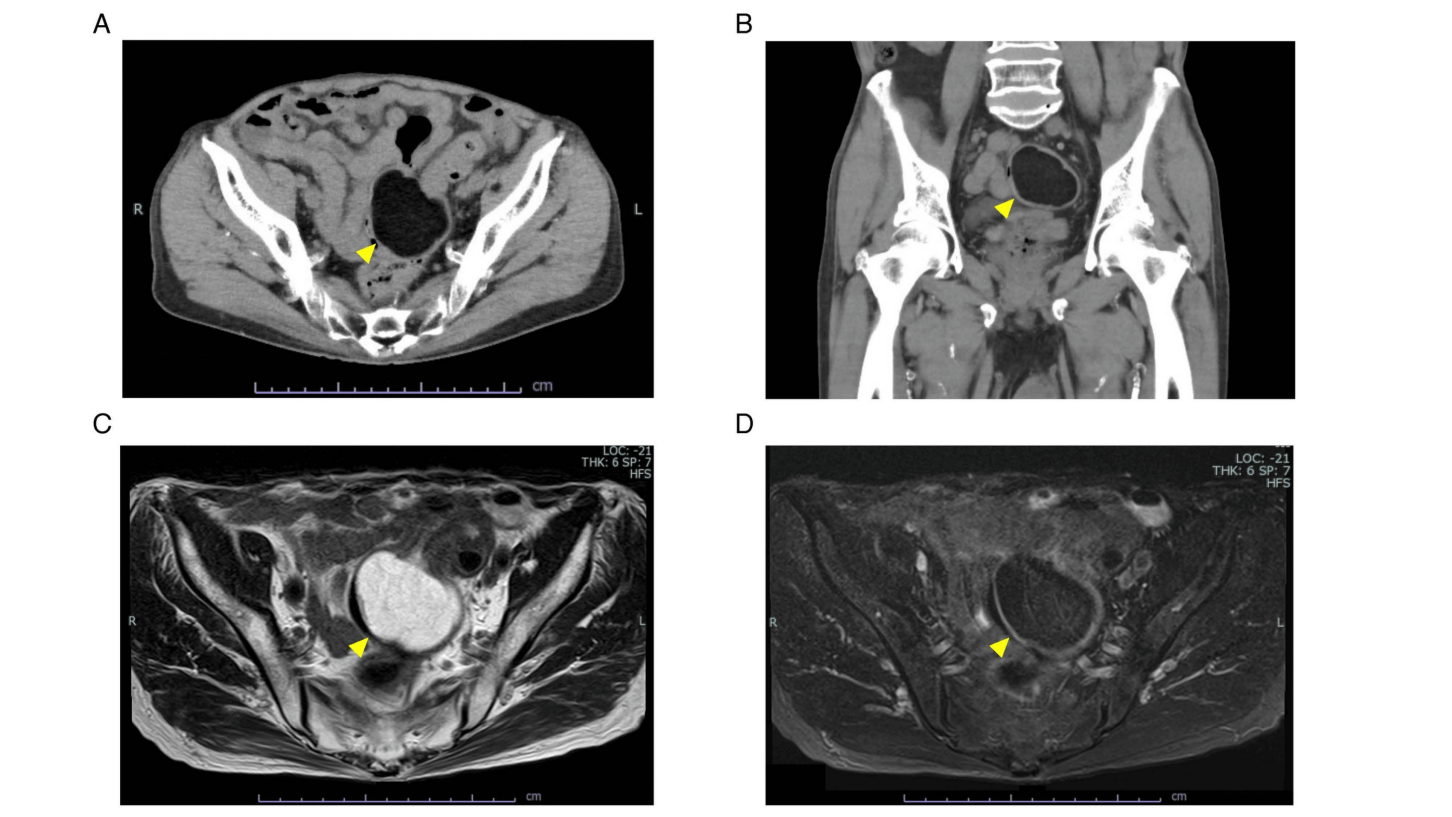
Preoperative imaging findings of the sigmoid colonic lipoma (A, B) CT images reveal a well-demarcated, low-density lesion in the sigmoid colon, consistent with adipose tissue. (C, D) MRI further confirms the lipomatous nature of the lesion with high signal intensity on T1-weighted images and fat suppression. Arrowheads indicate the tumor in all panels (A–D).

Preoperative biopsy from the erosion showed granulation tissue with inflammation but no signs of malignancy. The erosion at the top of the tumor was considered the cause of the bleeding, exacerbated by his anticoagulant medication. Consequently, he decided on an elective operation to alleviate the persistent symptoms. Preoperative evaluation using CT and MRI revealed findings consistent with a lipoma, while the size was almost unchanged from the initial MRI (Figure 2).

**Figure 3:**
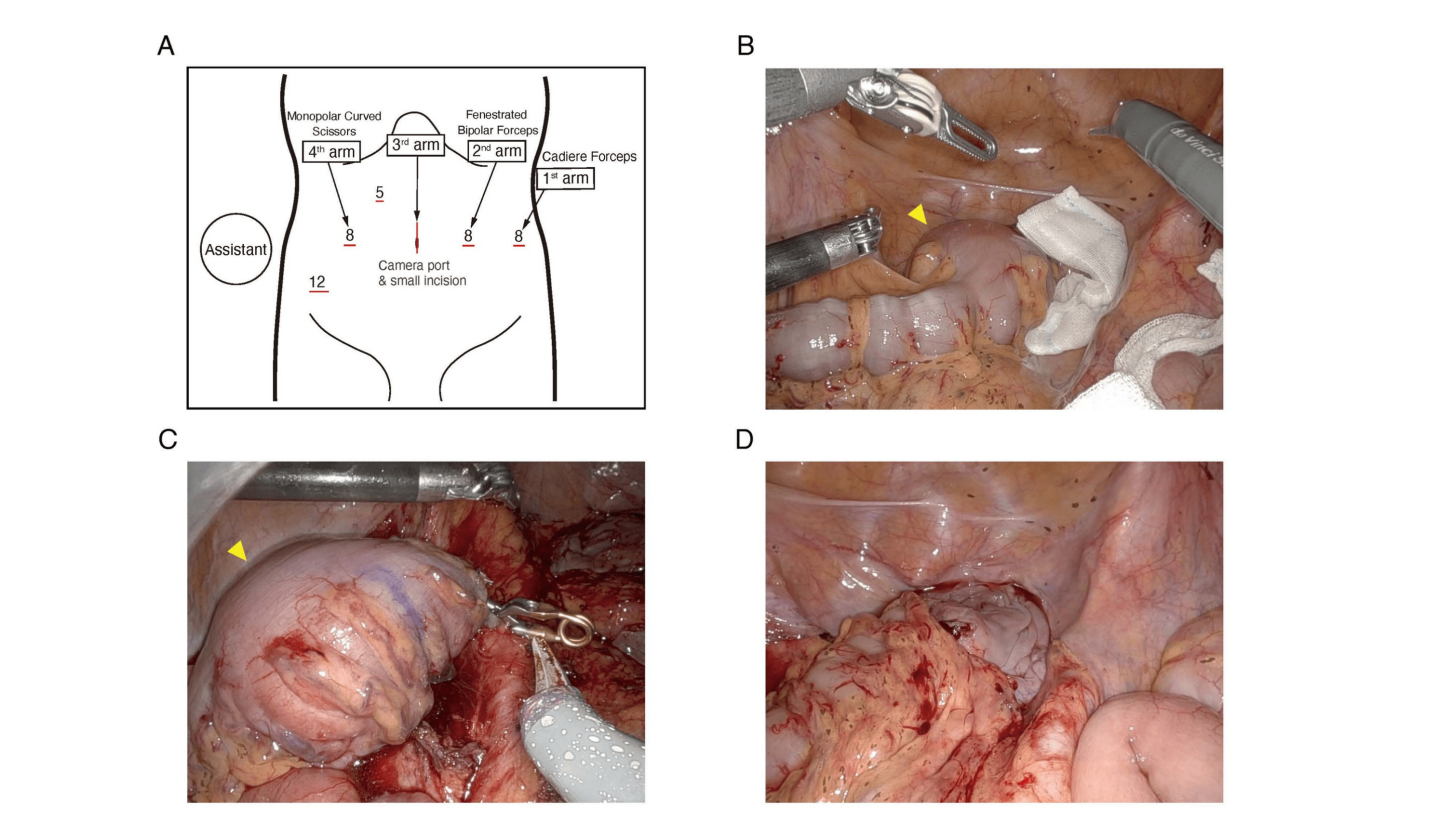
Operative setup and key steps of the robotic-assisted sigmoid resection (A) Trocar placement, including a small periumbilical incision for EZ access and additional robotic ports. (B) Medial approach dissection, preserving the hypogastric nerve while dissecting the superior rectal and sigmoid arteries (arrowhead indicates the tumor). (C) Rectal transection using a 60-mm stapler (arrowhead indicates the tumor). (D) Reconstruction with a double-stapling technique using a 29-mm circular stapler.

One month after the last bleeding event, robotic-assisted sigmoid resection was performed following standard surgical principles for sigmoid colon cancer resection. The robotic approach utilizing the DaVinci Xi Surgical System (Intuitive Surgical, CA, USA) was chosen to facilitate precise dissection, although laparoscopic resection was also feasible. He was positioned in a 20° Trendelenburg supine position. A small periumbilical incision was made for EZ access (Hakko Co., Ltd., Nagano, Japan) and camera port placement (Figure 3). In addition to four DaVinci ports, two assistant ports (12 mm and 5 mm) were placed according to the reported technique [8].

**Figure 4:**
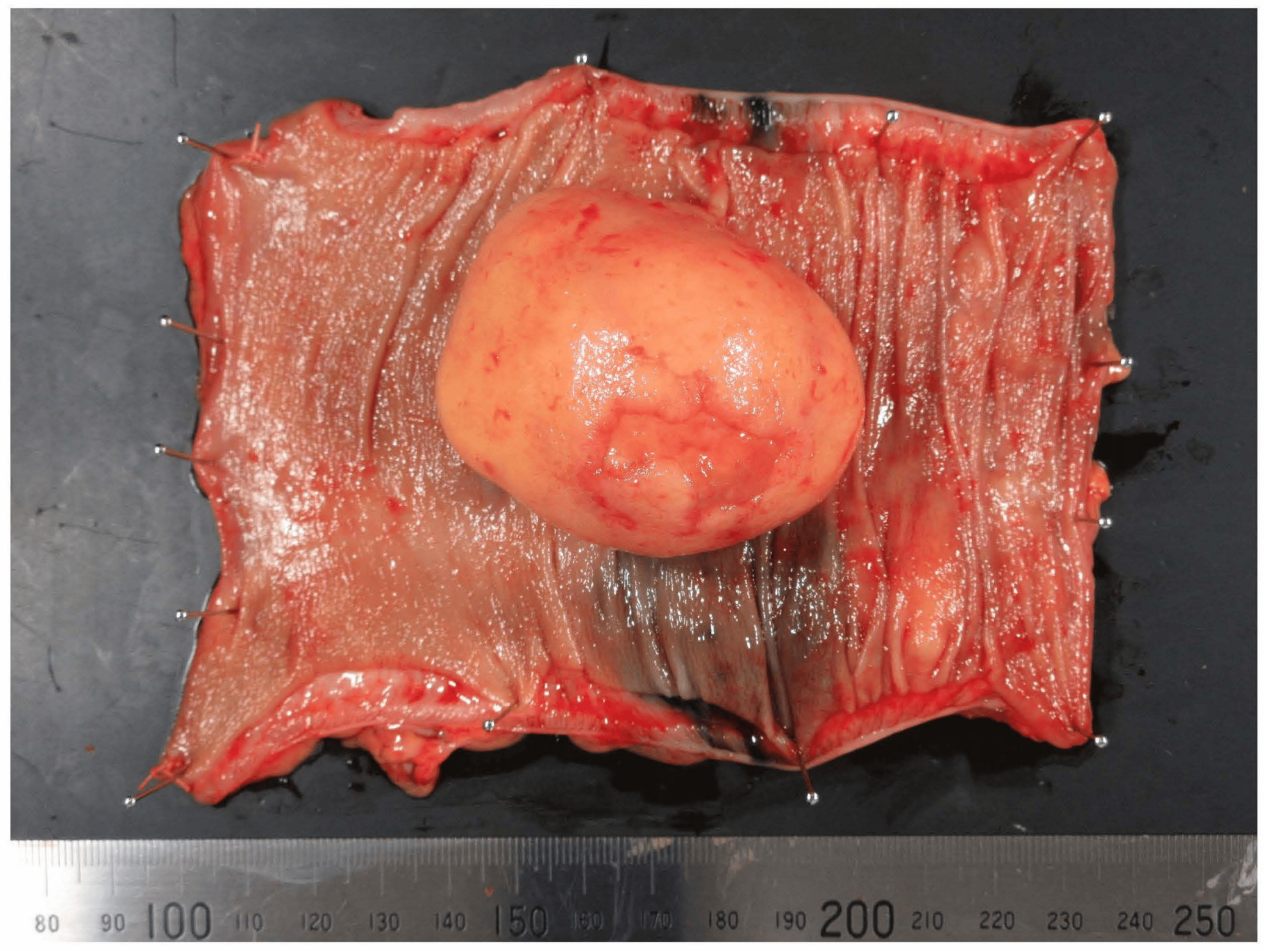
Macroscopic view of the resected specimen The tumor is a well-encapsulated, soft, yellowish mass originating from the submucosal layer. No malignant features are observed.

**Figure 5:**
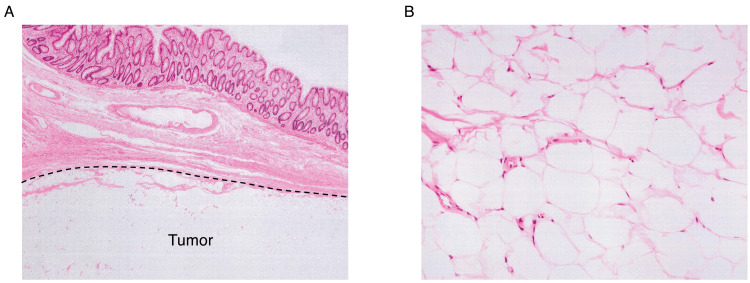
Histopathological examination of the resected sigmoid colonic lipoma (A) Hematoxylin and eosin (H&E) staining shows mature adipose tissue without cellular atypia, confirming the diagnosis of a benign lipoma. (B) The lesion is well-demarcated, with no evidence of malignancy or infiltration into the muscularis propria.

Intraoperatively, the giant sigmoid CL was easily identified. The procedures were initiated using a medial approach, focusing on the avascular plane in the posterior space to preserve the hypogastric nerve. For the vessel dissection, the left colic artery was preserved to maintain perfusion to the descending colon. The superior rectal artery and the sigmoid artery were clipped and dissected, and the inferior mesenteric vein was dissected at the same level. No lymphadenectomy was performed. The inter-sigmoid recess was opened, and the dissection was switched to a lateral approach. After complete mobilization of the sigmoid colon and dissection of the posterior rectal space, the rectum was transected using a 60-mm stapler. Reconstruction was performed with the double-stapling technique with a 29-mm circular stapler, and a drain was placed behind the anastomosis. The operation time was 246 minutes, with minimal bleeding and no complications. Pathological analysis of the resected specimen revealed lipoma cells from the sub-mucosal layer, with no evidence of malignancy (Figures 4, 5).

He was discharged from our hospital two weeks after surgery with no major complications and has been followed up in an outpatient setting. Eight months after surgery, no recurrence has been observed, and the patient's bowel function has returned to normal. Given that CLs are benign and complete resection was confirmed, no long-term oncologic surveillance was required.

## Discussion

Lipomas can develop throughout the body, from the hypopharynx to the rectum, with a rare intestinal occurrence [6,9]. CLs are the third most common benign colonic lesion after hyperplastic and adenomatous polyps, accounting for 1.8% of all cases [1,10]. They originate from the submucosa in approximately 90% of cases and the subserosa in the remaining 10%, with their size ranging from 2 mm to 30 cm [1,5,10]. While most CLs are asymptomatic and discovered incidentally, symptoms occur in approximately 6-25% of cases, with abdominal pain being the most frequent, followed by constipation, hematochezia, and diarrhea. Lesions exceeding 2 cm in size - typically those ≥4 cm - may cause bowel obstruction, intussusception, bleeding, or perforation, necessitating intervention [2,3,9,11,12]. Lipomas exceeding 5 cm in diameter are classified as “giant lipomas,” with 75% presenting with symptoms, and the majority (88%) of CLs causing colocolic intussusception fall into this category [2,12].

Endoscopically, CLs are usually observed as well-defined, soft, round or ovoid, yellowish lesions that may be sessile or pedunculated, although ulcerations and erythema may occasionally occur, potentially mimicking malignancy [2,13]. Abdominal CT scanning is the most commonly used diagnostic modality for CLs, detecting uniformly low-attenuation lesions (-40 to -120 Hounsfield units), which have a sensitivity of 71-87% and a specificity of up to 100% [2,4,12,14]. MRI also provides valuable diagnostic indications, particularly in cases of large CLs. Due to the unique signal characteristics of adipose tissue, MRI effectively highlights these lesions, especially on T1-weighted and fat-suppressed images, facilitating differentiation from other soft tissue tumors [4,12].

The management of CLs depends on their size, location, symptoms, presence of complications, presence or absence of preoperative diagnosis, and morphology (sessile or pedunculated) [2]. Small (<2 cm) asymptomatic lipomas generally require no intervention and can be monitored; however, symptomatic lipomas or those exceeding 2 cm in size should be considered for resection [13]. Endoscopic resection is particularly suited for smaller, superficial lesions, with 2 cm generally considered the upper limit to avoid the risk of perforation [4]. In the present case, the giant lipoma itself obstructed visualization of its base, making endoscopic treatment challenging.

Surgical resection is the standard treatment for large CLs, particularly those exceeding 2 cm, involving the muscular or serosal layers, or complicated by intussusception or obstruction [4,12,15]. For lower rectal lipomas with anal protrusion, transanal local excision remains a viable and favored option if feasible [16,17]. An abdominal approach is most commonly employed for colonic lesions, with surgical options including colotomy with local excision (enucleation), limited colon resection, segmental resection, and hemicolectomy [1,4]. The former procedures are indicated when the preoperative diagnosis is definitive, whereas the latter are recommended when the diagnosis remains uncertain or complications such as an intussusception or bowel obstruction occur, especially when malignancy cannot be ruled out [4,18]. Laparoscopy and robotic-assisted surgery are preferred for CL resection due to reduced pain, shorter hospital stays, and faster recovery compared to traditional open surgery [12,19-21]. However, precise identification of lipomas can be challenging [19], for which endoscopic clip placement or tattooing may help [1]. Most minimally invasive surgery for CLs has been laparoscopic; to our knowledge, this is the first reported robotic-assisted sigmoidectomy for a CL, while robotic-assisted resection of pelvic mesorectum lipoma [22] and CLs in other parts of the colon has been described [23,24]. Given its established role in colorectal cancer, a robotic approach is feasible for CLs and may facilitate precise dissection through articulated instruments, tremor filtering, three-dimensional vision, and a stable operative field [7]. While laparoscopy is standard, reports in diverticulitis indicate that robotic colectomy achieves similar morbidity with lower conversion rates and a slightly shorter length of stay, though at the expense of longer operative time and higher costs [25]. Our operative time (246 minutes) exceeded these typical values (~200 minutes), reflecting the robotic learning curve. The surgical approach should be individualized according to lipoma size, location, comorbidities, and the surgeon’s expertise and procedural feasibility. As in our case, CLs have an excellent prognosis, with no documented recurrence following complete resection [13].

## Conclusions

We report a rare surgical case of a giant lipoma located in the sigmoid colon. CLs larger than 2 cm that become symptomatic warrant surgical resection. Robot-assisted partial colectomy was safely performed in our case and can be a feasible surgical option for CLs.
